# Sea Turtle Population Genomic Discovery: Global and Locus-Specific Signatures of Polymorphism, Selection, and Adaptive Potential

**DOI:** 10.1093/gbe/evz190

**Published:** 2019-09-04

**Authors:** Julie C Chow, Paul E Anderson, Andrew M Shedlock

**Affiliations:** 1 Integrative Genetics and Genomics Graduate Group, University of California, Davis; 2 Department of Computer Science, College of Charleston, Charleston, South Carolina; 3 Department of Biology, College of Charleston, Charleston, South Carolina; 4 College of Graduate Studies, Medical University of South Carolina; 5 Marine Genomics Division, Hollings Marine Laboratory, Charleston, South Carolina; 6 Department of Computer Science and Software Engineering, California Polytechnic State University, San Luis Obispo, CA 93407

**Keywords:** single-nucleotide polymorphism, loggerhead, genotyping-by-sequencing, linkage disequilibrium, temperature-dependent sex determination

## Abstract

In the era of genomics, single-nucleotide polymorphisms (SNPs) have become a preferred molecular marker to study signatures of selection and population structure and to enable improved population monitoring and conservation of vulnerable populations. We apply a SNP calling pipeline to assess population differentiation, visualize linkage disequilibrium, and identify loci with sex-specific genotypes of 45 loggerhead sea turtles (*Caretta caretta*) sampled from the southeastern coast of the United States, including 42 individuals experimentally confirmed for gonadal sex. By performing reference-based SNP calling in independent runs of *Stacks*, 3,901–6,998 SNPs and up to 30 potentially sex-specific genotypes were identified. Up to 68 pairs of loci were found to be in complete linkage disequilibrium, potentially indicating regions of natural selection and adaptive evolution. This study provides a valuable SNP diagnostic workflow and a large body of new biomarkers for guiding targeted studies of sea turtle genome evolution and for managing legally protected nonmodel iconic species that have high economic and ecological importance but limited genomic resources.

## Introduction

Turtles are among the most iconic of vertebrate lineages, yet the genomic basis for their unique adaptive evolutionary biology is just starting to be investigated with modern high-throughput DNA diagnostics, especially those species that have evolved fully pelagic marine life histories. Global sea turtle populations exhibit remarkable longevity, fecundity, physiological versatility, and astonishing migratory capabilities that have collectively gained them a reputation for being highly resilient persistent species (Duchene et al. 2012; Novelletto et al. 2016), however, their survival is increasingly threatened by fisheries by-catch, coastal development, human consumption, and climate change (Lewison, Crowder, et al. 2004; Lewison, Freeman, et al. 2004; Fish et al. 2005; Hawkes et al. 2007; Witt et al. 2010). Sea turtles maintain seagrass bed and coral reef health and influence ocean nutrient cycling, productivity, and biodiversity (Orth et al. 2006; Goatley et al. 2012). Most species of sea turtle share similar lifecycles involving a juvenile pelagic phase and subadult neritic developmental phase (Luschi et al. 2003). Loggerhead sea turtles (*Caretta caretta*) are found in the Atlantic, Pacific, and Indian Oceans, with major loggerhead rookeries spanning from southwest Florida to North Carolina (Witherington et al. 2009). Juvenile loggerheads migrate from rookeries and begin a new developmental stage in oceanic gyres for several years (Carr 1987; Bjorndal et al. 2001). Subadult loggerheads may spend their lives feeding on oceanic waters or may relocate to neritic zones and feed on benthic prey until maturation, after which adult loggerheads return to natal colonies to reproduce (Bowen et al. 2005; McClellan and Read 2007). Loggerhead sea turtles are listed as vulnerable by the IUCN Red List of Threatened Species (http://redlist.org; last accessed September 12, 2019). Recent research indicates that sea turtle populations may be at further risk due to increased proportion of females attributed to temperature-dependent sex determination (TSD) and rising global temperatures (Jensen et al. 2018). The identification of DNA markers, particularly of loci with sex-specific genotypes, and estimation of population structure and genetic diversity via the extent of genome-wide linkage disequilibrium will benefit the conservation of future sea turtle populations.

Advances in next-generation sequencing (NGS) technologies have revolutionized the detection of genetic variation and signatures of selection within populations (Slatkin 2008; Davey et al. 2011). Single-nucleotide polymorphisms (SNPs) have emerged as molecular markers of choice due to their high abundance and density, low mutation rate, and normally biallelic nature (Vignal et al. 2002; Brumfield et al. 2003; Morin et al. 2004). The reliability and relatively cheap cost of SNPs in comparison to genotyping methods such as microsatellites (short tandem repeats) have made SNPs an important tool to calculate population genetics statistics and identify patterns of adaptation (Namroud et al. 2008; Beissinger et al. 2013). Reduced representation libraries (RRLs) reduce genome complexity and permit SNP calling without requiring whole genome sequencing (Altshuler et al. 2000; Pootakham et al. 2015). Among reduced representation approaches, restriction-associated DNA sequencing and genotyping-by-sequencing (GBS) are restriction enzyme-based methods used to discover a high density of SNP markers (Miller et al. 2007; Baird et al. 2008; Elshire et al. 2011; Poland et al. 2012; Andrews et al. 2016). Multiplex sequencing of restriction enzyme-associated sites is cost effective, increases copy numbers of nucleotides adjacent to restriction sites, avoids repetitive genomic regions, and allows high-throughput SNP discovery (Elshire et al. 2011; Beissinger et al. 2013).

We apply a SNP calling pipeline to 45 loggerhead sea turtles (*C.caretta*) sampled from the southeastern United States in compliance with authorized federal and state agency permitting and established tissue collection operations. To describe global and loci-specific signatures of natural selection, we use GBS to call thousands of high-quality SNPs, estimate population structure and linkage disequilibrium, identify potential sex-specific loci, and functionally annotate identified SNPs. Other endangered populations for which there are limited genomic resources can benefit from the outlined procedure that facilitates understanding of the adaptive potential of populations and can aid future conservation efforts (Eizaguirre and Baltazar-Soares 2014).

## Materials and Methods

### Population Sampling and DNA Extraction

**Fig. 1.**
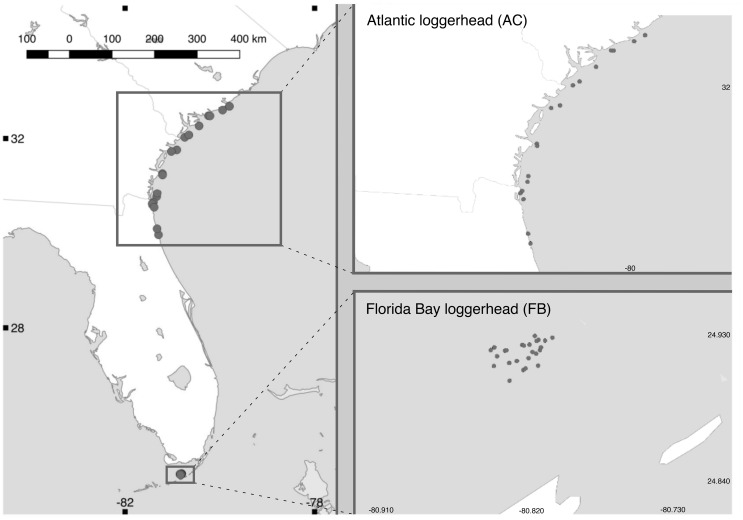
—Geographic locations of Atlantic (AC) and Florida Bay (FB) loggerhead sea turtles.

Loggerhead sea turtles were sampled from the U.S. Atlantic Coast and Florida Bay, Everglades National Park (fig. 1). Turtles sampled from the Atlantic Coast ranged in carapace length from 44.7 to 77.9 cm and were collected using commercial fishing gear from 21 established marine sampling stations off the coasts of Florida, Georgia, and South Carolina from May to August 2014–2015 for the South Carolina Department of Natural Resources In-water Sea Turtle Populations Surveys (http://www.dnr.sc.gov/marine/sturtles/methods.html; last accessed September 12, 2019). Florida Bay turtles were captured from the Everglades National Park in June 2009 for the National Park Service marine turtle inventory (David Owens, College of Charleston Grice Marine Laboratory). Sex was determined by testosterone radioimmunoassay (Braun-McNeill et al. 2007) and laparoscopic gonadal inspection in Atlantic Coast and Florida Bay sea turtles, respectively (Owens et al. 1978).

From each sea turtle, a minimum of 25 ml of whole blood was extracted from the dorsal cervical sinus into BD Vacutainer sterile red-top clinical collection tubes (BD Biosciences, San Jose, CA) and immediately frozen at −20 °C (Owens and Ruiz 1980). To extract DNA, 20 µl of whole blood was incubated with 180 µl phosphate buffered saline, 20 µl Proteinase K, and 200 µl Qiagen buffer AL for 1 h. The solution was transferred to a Qiagen DNEasy Blood and Tissue Kit spin column (Qiagen, Redwood City, CA), and sample concentrations were measured with a Qubit fluorometer (Life Technologies, Grand Island, NY) following the manufacturer’s protocol. Sampled individuals were divided into two groups based on location of capture, referred to as Florida Bay (FB) and Atlantic loggerheads (AC) (fig. 1 and table 1).

**Table 1:** Sampling Locations and Reads Used from *Ch. mydas* Reference-Based SNP Calling

Individual	Sex	Long. (–)	Lat.	Number of Loci	Reads Used	Coverage
AC0705	F	80.742	32.024	1,182,653	1,521,517	1.441
AC0706	M	80.7654	32.073	1,799,804	2,641,903	1.693
AC0707		80.439	32.265	1,228,560	1,605,876	1.466
AC0708	F	80.242	32.479	1,365,611	1,806,373	1.492
AC0709	F	80.209	32.475	924,921	1,137,505	1.361
AC0710	F	79.942	32.598	1,557,346	2,156,585	1.579
AC0712	F	79.799	32.677	1,109,024	1,411,227	1.418
AC0713	F	79.798	32.677	247,696	270,026	1.163
AC3135	M	80.909	31.760	1,324,309	1,736,182	1.469
AC3136	F	81.025	31.726	1,893,336	2,759,151	1.667
AC3141	M	81.212	31.260	2,101,417	3,257,078	1.792
AC3142	M	81.210	31.232	1,178,400	1,498,117	1.415
AC3143	M	81.210	31.232	684,402	802,777	1.279
AC3146		81.334	30.763	939,074	1,142,528	1.344
AC3147	M	81.403	30.644	771,794	924,967	1.316
AC3148	M	81.430	30.613	1,920,393	2,828,535	1.692
AC3149	M	81.388	30.537	1,567,760	2,163,188	1.564
AC3150	M	81.296	29.958	1,632,693	2,305,614	1.601
AC3151	M	81.326	30.085	1,373,303	1,818,685	1.494
AC3155		81.323	30.837	1,561,533	2,172,262	1.576
1FB	M	80.813	24.911	744,535	890,241	1.304
FB10	M	80.834	24.921	2,044,700	3,201,311	1.819
FB11	M	80.833	24.921	1,326,160	1,776,573	1.506
FB12	M	80.822	24.909	1,353,237	1,800,683	1.494
FB13	M	80.804	24.929	1,267,15	1,651,180	1.459
FB14	M	80.839	24.917	1,187,114	1,533,697	1.446
FB15	M	80.823	24.924	1,048,180	1,312,212	1.39
FB29	F	80.821	24.910	1,424,617	1,893,274	1.496
FB31	F	80.812	24.921	630,034	731,210	1.263
FB32	F	80.813	24.927	1,882,617	2,746,099	1.673
FB33	F	80.819	24.916	1,038,976	1,300,666	1.389
FB35	F	80.809	24.927	1,574,431	2,199,361	1.583
FB36	F	80.815	24.930	1,188,247	1,526,448	1.426
FB37	F	80.826	24.914	1,584,775	2,194,197	1.562
FB38	F	80.841	24.911	1,254,056	1,623,560	1.45
FB4	M	80.814	24.919	1,054,494	1,339,609	1.409
FB41	F	80.819	24.924	1,756,596	2,590,986	1.689
FB43	F	80.822	24.924	1,972,914	2,962,513	1.735
FB44	F	80.812	24.923	1,346,055	1,788,969	1.495
FB49	F	80.814	24.920	1,598,288	2,198,554	1.554
FB5	M	80.817	24.920	2,086,740	3,234,278	1.801
FB6	M	80.843	24.921	1,321,940	1,741,413	1.48
FB7	M	80.831	24.913	478,067	545,503	1.235
FB8	M	80.840	24.923	2,463,364	4,244,613	2.044
FB9	M	80.828	24.902	1,138,391	1,449,918	1.416

Note.—Longitude (long.) and latitude (lat.) are displayed in degrees for Atlantic (AC) and Florida Bay (FB) loggerhead sea turtles. Number of loci, number of forward reads used, and weighted mean coverage (such that coverage at shared loci is weighted more heavily) are shown for individuals for which at least one locus was retained following genotype quality filtering.

### GBS Preparation and Sequencing

GBS libraries were prepared in cooperation with the Clemson University Genome Institute (www.clemson.edu/centers-institutes/itg; last accessed September 12, 2019) according to the procedure developed for high diversity maize genomes by Elshire et al. (2011) using the methylation-sensitive restriction enzyme *Ape*KI. We designed barcodes of variable length for multiplex sequencing (supplementary table 1, Supplementary Material online). Cycles (2-by-125) of paired-end sequencing was performed on 2 lanes of the Illumina HiSeq 2500 v.4.0 NGS diagnostics platform according to standard manufacturer protocols in the Center for Genomic Medicine at the Medical University of South Carolina (www.hollingscancercenter.org; last accessed September 12, 2019).

### Reference-Based SNP Calling and Quality Filtering

To demultiplex and clean raw reads, we used the script “process_radtags.pl” from the program *Stacks* (version 2.2) (Catchen et al. 2011, 2013). For reference-based SNP calling, we aligned processed reads to *Chelonia mydas* (version 1.0; GCA_000344595.1) and *Chrysemys picta bellii* (version 3.0.3; GCA_000241765.2) genome assemblies using BWA-MEM and performed SNP calling via the *gstacks* program while requiring base call accuracy to be >95% (–gt-alpha 0.05) (Li and Durbin 2009; Shaffer et al. 2013; Wang et al. 2013). The threshold to call genotypes (–gt-alpha 0.05) discards loci that are missing for many individuals, and thus affects analyses using individual genotypes, but does not affect analyses independent of coverage such as SNP identification and general diversity indices. The *populations* program of *Stacks* was used to calculate various population genetics statistics given “population maps” that defined all samples to be from the same group or two separate groups based on capture location: Atlantic *C. caretta* (AC) and Florida Bay *C. caretta* (FB). SNP calls were filtered to require that a locus be present in a minimum of 75% of individuals within a group (-*r* 0.75), have a minor allele frequency of at least 0.01 (–min_maf 0.01), and have a heterozygosity <0.70 (–max_obs_het 0.70) to reduce paralogs.

### Assessment of Loci with Sex-Specific Genotypes, Linkage Disequilibrium, and Population Differentiation

To explore potential sex-specific genotypes from sexed turtles, we calculated the probability that observed allele frequencies occurred in sexed turtles via Fisher’s exact test with false discovery rate correction (Genepop version 4.7) (Raymond and Rousset 1995; Rousset 2008) and estimated the extent of genome-wide linkage disequilibrium via PLINK (version 1.07) and Haploview (version 4.2) (Barrett et al. 2005; Purcell et al. 2007). We filtered loci to require at least a minor allele frequency of 0.05 (-minMAF 0.05), excluded markers with <0.80 nonzero genotypes (-minGeno 0.80) and Hardy Weinberg *P* values <0.001 (-hwcutoff 0.001), excluded individuals with more than 0.80 missing data (-missingCutoff 0.80), and noted the distance (in basepairs) between loci and the number of pairs of loci that display an *r*-squared value equal to 1, *D*′ > 0, or significantly associated loci pairs with log odds ratio (LOD) > 2. Due to the possibility of strong linkage disequilibrium existing simply due to short distance between loci pairs, we consider loci pairs at a minimum distance of 250 basepairs to better infer the potential effects of selection. The *populations* parameter option *–write_single_snp* was enabled for *STRUCTURE* (version 2.3.4) analysis to select only the first SNP in a locus to ensure SNPs are unlinked. The program *STRUCTURE* was used to describe population structure with estimated number of clusters (*K*) varied from 2 to 5 with burnin length of 10,000 and 20,000 iterations (Hubisz et al. 2009), and admixture proportions were visualized with the R package *pophelper* (Francis 2017). Optimal *K* was estimated as the *K* producing the greatest delta *K* with the Evanno method via *pophelper* with ten replicates per *K.* Identified variants were functionally annotated with ANNOVAR (version April 16, 2018) (Wang et al. 2010) with *Chr. picta* as a reference and associated annotated gene and protein files (Shaffer et al. 2013; Wang et al. 2013).

## Results

### Sequencing and SNP Detection

GBS libraries produced ∼308,077,794 raw reads for 45 loggerhead sea turtles, and 304,188,286 reads were retained following “*process_radtags*” filtering (supplementary table 1, Supplementary Material online). Following “*process_radtags*” filtering, an average of 1,876,381 reads were used per individual at 1.5× coverage using *Ch. mydas* as a reference (table 1 and supplementary table 2, Supplementary Material online), and similar values were observed for *Chr. picta* reference-based SNP calling (supplementary table 3, Supplementary Material online). Reference-based calling methods using *Ch. mydas* produced the greatest number of filtered loci (24,616–119,653) and SNPs (5,018–6,998) called compared with *Chr. picta* reference-based method with consideration of sea turtles as either a single group or two separate groups for population genetics analyses (table 2). For all identified variants, all were annotated as intergenic and >1 kb away from a gene via ANNOVAR (supplementary tables 4–7, Supplementary Material online).

**Table 2:** Filtered Loci and Variant Sites (SNPs) Identified via Reference-Based Methods with Varied Group Assignment

	Number of Groups	SNPs	Loci
*Ch. mydas*	1	5,018	24,616
	2	6,998	119,653
*Chr. picta*	1	3,901	20,666
	2	5,234	69,838

Note.—Atlantic (AC) and Florida Bay (FB) loggerhead sea turtles were considered as either a single group or two separate groups during populations analyses.

### Estimation of Genetic Diversity

**Fig. 2.**
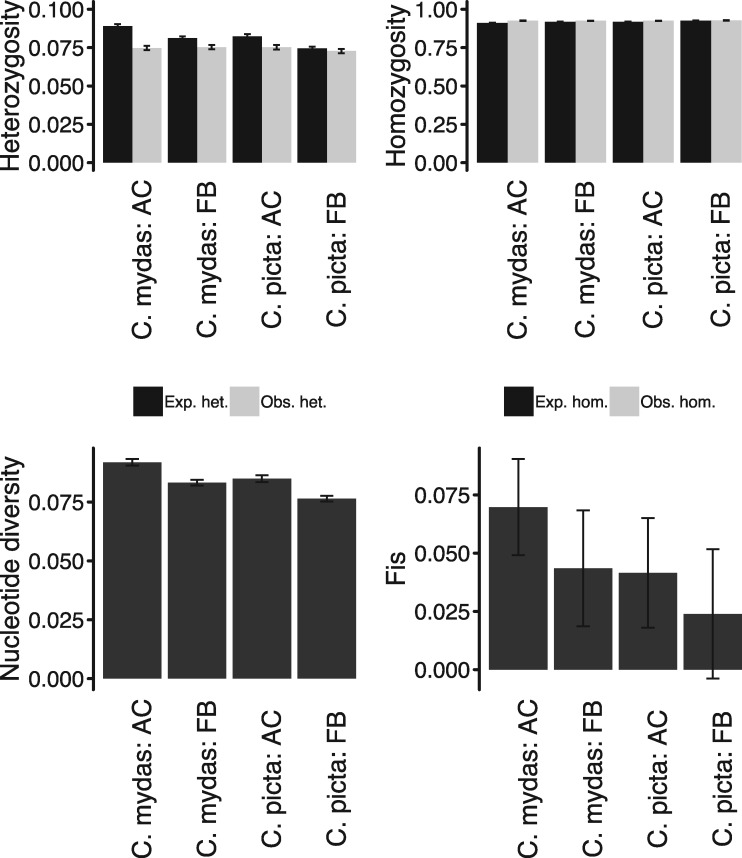
—Population genetics statistics. Mean observed and expected heterozygosity and homozygosity, nucleotide diversity, and inbreeding coefficients are displayed for variant sites with standard error bars for Florida Bay (FB) and Atlantic (AC) loggerheads using either *Ch. mydas* or *Chr. picta* as a reference during SNP calling.

*F*-statistics were calculated to assess genetic diversity of Atlantic and Florida Bay loggerheads as two distinct groups or a single group (Wright 1951). Fixation indices (*F*_ST_) among AC and FB groups reveal that population differentiation is limited and ranges from 0.0137 (*Ch. mydas* as reference) to 0.0141 (*Chr. picta* as reference), differing significantly according to Wilcoxon signed rank tests. Observed homozygosity at variant sites is relatively similar in FB and AC loggerheads, and nucleotide diversity (*π*) (Nei and Li 1979) tends to be greater in AC loggerheads (fig. 2 and supplementary table 8, Supplementary Material online). Generally, the use of either *Ch. mydas* or *Chr. picta* as a reference during SNP calling revealed similar trends and comparable measures in population genetics statistics. Additionally, *Ch. mydas* and *Chr. picta* reference-based calls yield small (<0.07), nonzero inbreeding coefficients (*F*_IS_) at variant positions and “all” (variant and nonvariant) positions, indicating a greater excess of homozygotic calls found when considering AC and FB loggerheads as separate groups rather than a single group. Nucleotide diversity is decreased and observed homozygosity is increased for calls produced without distinguishing AC and FB individuals into two groups.

### Determination of Sex-Specific Loci and Extent of Linkage Disequilibrium

**Fig. 3.**
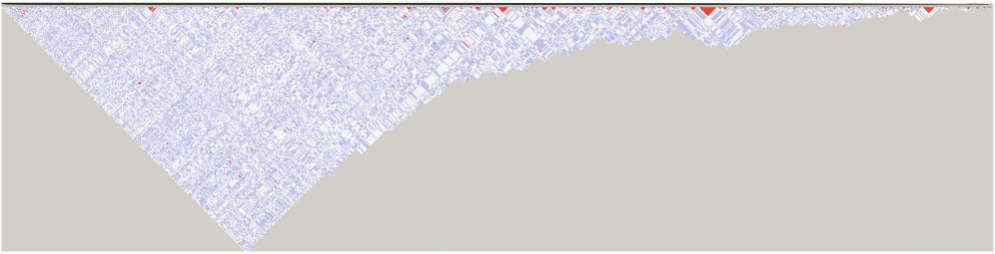
—Linkage disequilibrium (LD) plot of loci produced via Haploview for *Ch. mydas* reference-based SNP calling and consideration of Florida Bay (FB) and Atlantic (AC) loggerheads as separate groups. Color indicates *D*′ and LOD values (white: *D*′ < 1 and LOD < 2; blue: *D*′ = 1 and LOD < 2; shades of pink/red: *D*′ < 1 and LOD ≥ 2; bright red: *D*′ = 1 and LOD ≥ 2). Numbers within squares represent *D*′ × 100, and unnumbered squares have *D*′ = 1.

Hundreds of pairs of loci were found to be in complete linkage disequilibrium (*r*^2^ = 1), display *D*′ > 0, or were significantly associated with a log odds score (LOD) of at least 2 (fig. 3 and supplementary fig. 1 and supplementary table 9, Supplementary Material online). For SNP calling methods that considered AC and FB as a single group, ∼18–69 pairs of loci at least 250 bp apart display *r*^2^ = 1 and LOD > 2. Consideration of AC and FB as two separate groups estimates 17–68 pairs of loci at least 250 bp apart to be in complete disequilibrium with LOD > 2. Overall, SNP calls produced by using *Ch. mydas* as opposed to *Chr. picta* as a reference identified a greater number of loci with significant linkage disequilibrium. According to Fisher’s exact test, sexed samples were identified as having 29 (*Chr. picta* as a reference) and 30 (*Ch. mydas* as a reference) loci with sex-specific genotypes according to Fisher’s exact test (*q-value* <0.05, supplementary tables 10 and 11 and supplementary fig. 2, Supplementary Material online).

### Distinct Population Structure in Atlantic Loggerheads

**Fig. 4.**
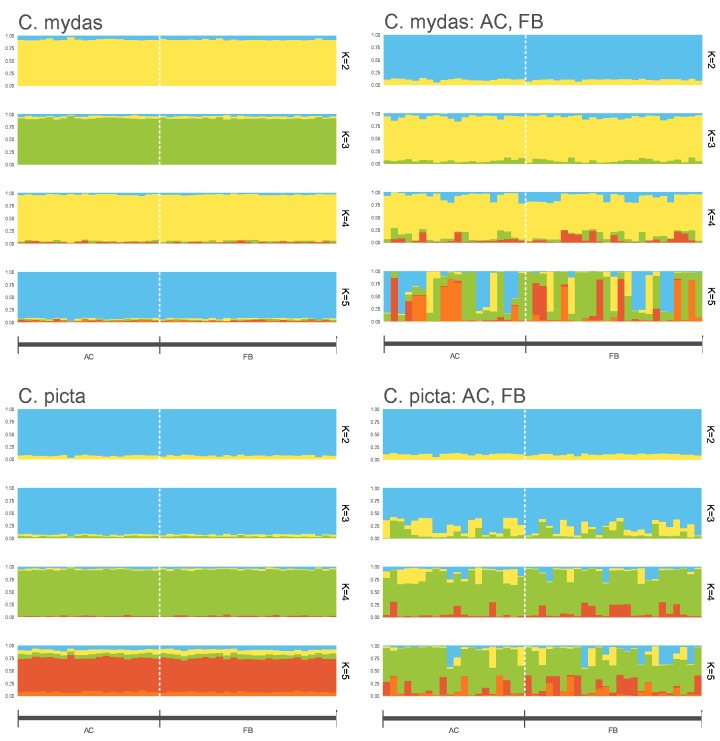
—Population structure with varied number of assumed populations (*K*) using *Ch. mydas* or *Chr. picta* as a reference during SNP calling. Atlantic (AC) and Florida Bay (FB) loggerhead sea turtles were considered as a single group or as two separate groups (AC, FB) during population analyses.

To describe population structure in loggerheads, we varied the number of clusters or populations for *K* from 2 to 5 with 10 replicates per *K* and observed the admixture proportion of each individual (fig. 4). With consideration of AC and FB individuals as a single group, admixture plots show little differentiation between AC and FB individuals. The highest delta *K* determined via the Evanno method indicated that the optimal *K* using either *Ch. mydas* or *Chr. picta* as a reference with consideration of AC and FB individuals as separate or a single group was *K = *2. Using *Chr. picta* or *Ch. mydas* as a reference, at *K *=* *2, most individuals show more than 80% admixture from a single cluster. Most AC and FB individuals show similar patterns of admixture, again indicating detection of relatively little population differentiation. As *K* increases, admixture plots using SNP calls that had separately grouped AC and FB individuals indicate that almost all individuals can be assigned to one of *K* clusters. Additionally, multidimensional scaling reveals clustering and decreased genetic distance among FB individuals relative to AC individuals (supplementary fig. 3, Supplementary Material online).

## Discussion

We describe the application of a SNP calling pipeline to Atlantic loggerhead sea turtles that identifies loci in linkage disequilibrium and potentially sex-specific loci, calculates population genetics statistics, and assesses population structure. Reference-based SNP discovery has the potential to identify more SNPs than de novo methods; greater relatedness of the reference to the focal species tends to result in a greater number of SNPs called (Burford Reiskind et al. 2016). *Cheloniamydas*, the green sea turtle, is more closely related to loggerhead sea turtles than is *Chr. picta*, the western painted turtle, and the use of *Ch. mydas* as a reference identifies 1,117–1,764 more SNPs than using *Chr. picta* as a reference (table 2). The use of low-depth GBS and stringent missingness filters presented here offers an initial examination of loggerhead genetic diversity. Due to low mean coverage observed in Atlantic (AC) and Florida Bay (FB) loggerhead sea turtles, the ability to call genotypes and distinguish heterozygotes and homozygotes is compromised, thus requiring genotype quality filtering. Analyses involving individual genotypes, such as the identification of loci with sex-specific genotypes, loci in linkage disequilibrium, and admixture analysis are affected by coverage. Thus, analyses involving individual genotypes must be interpreted with caution due to low coverage resulting in decreased ability to confidently detect heterozygotes. Conversely, analyses independent of individual genotypes, such as SNP identification, general diversity indices, and *F*_ST_ estimates are independent of coverage. Using *Ch. mydas* as a reference and with consideration of AC and FB loggerhead sea turtles as either a single group or two groups during population genetics analyses, 20,666–119,653 loci and 3,901–6,998 SNPs were identified, in comparison to previous studies which have identified <100 haplotypes and have relied on fewer than 50 microsatellite markers to study loggerhead populations (Carreras et al. 2006, 2011, 2018; Monzon-Arguello et al. 2008; Sanderlin et al. 2009; Garofalo et al. 2013; Shamblin et al. 2014; Rees et al. 2017; Clusa et al. 2018).

Average population genetics statistics are comparable to previous studies of sea turtle population genetics that used nuclear and mitochondrial DNA haplotype-based analyses (supplementary table 8, Supplementary Material online) (Hatase et al. 2002; Carreras et al. 2007; Shamblin et al. 2007; Monzon-Arguello et al. 2008; Garofalo et al. 2009; Shamblin et al. 2012, 2014). Generally, *Stacks populations* analyses indicate slightly lower genetic diversity and nucleotide diversity in FB compared with AC loggerheads, but it is likely that the sampled individuals are members of several breeding populations in an overlapping geographic region (Bowen et al. 2005). The admixture analysis described in this study provides a possible method to assign individuals to breeding areas. Relatively low genetic diversity from our genome-wide analysis of anonymous unlinked polymorphic loci invites expanded investigation for the potential effects of population demographics on genomic diversity. Observing an excess of homozygotes is not totally unexpected and could be related to sampling individuals from local subpopulations with different genetic signatures, thereby producing a Wahlund effect. Although population bottlenecks of historically exploited species may lead to reduced allelic diversity despite secondary re-expansion of effective population abundance (Garza and Williamson 2001; Hauser et al. 2002; Roman and Palumbi 2003; Okello et al. 2008; Alasaad et al. 2011), there is no evidence that historical nest harvests or commercial by-catch of sea turtles decimated the Atlantic coast populations to a degree that would be expected to create protracted impacts on allelic diversity (Arendt et al. 2012, 2013). Relatively low observed heterozygosity in loggerhead sea turtles estimated here warrant cautious interpretation of the genetic health of wild sea turtles despite large levels of observed local abundance. We anticipate that genome-wide sampling of millions of SNPs in a smaller number of individuals representing regional management units will continue to refine our view of population health and locus-specific adaptive resiliency of loggerhead resources. This more technically complex effort that requires both high-throughput DNA sequencing and high-performance computing is an emerging new standard that can complement and extend the value of less expensive, widespread geographic sampling of mtDNA haplotypes and microsatellites for many individuals worldwide and drive a baseline of genetic data essential for integrated conservation strategies.

Levels of linkage disequilibrium are affected by natural selection, genetic drift, gene flow, and changes in population size (Hill and Robertson 1966; Nei and Li 1973; Slatkin 2008). Linkage disequilibrium appears to be relatively rare in loggerhead genomes, as is expected from an indexed library preparation and the abundance of noncoding genomic regions relative to exonic regions (Elshire et al. 2011). The identification of nonrandomly associated loci in intergenic regions suggests potential locus-specific regions of natural selection related to local environmental adaptation (Slatkin 2008). The functions of intergenic regions are not immediately apparent but may be involved with promoter, enhancer, and other regulatory element activity (Nelson et al. 2004; Sanyal et al. 2012; Mifsud et al. 2015).

The development of new molecular markers is necessary for future population genomics analysis of sea turtles (Lee 2008). In this study, we perform reference-based SNP calling of loggerhead sea turtles, predict candidate SNPs, and identify potential loci with sex-specific genotypes. Although our results concerning loci with sex-specific genotypes, linkage disequilibrium, and admixture analyses must be interpreted with caution due to overall low depth of coverage, to our knowledge, the present analysis is among the first uses of high-resolution multilocus genotypes to statistically identify sea turtles by sex in a noninvasive manner that is completely independent of morphology, which has remained elusive for turtle biologists worldwide and can have broad utility for the study of TSD, sex ratios, sex-bias gene flow, survivorship, and social structure of wild populations. This is especially important in informing integrated noninvasive studies of sexually monomorphic hatchlings and juveniles that are vulnerable to the potential impacts of environmental change (Jensen et al. 2018; Lasala et al. 2018; Sifuentes-Romero et al. 2018). The coexistence of both TSD and loci with sex-specific genotypes in loggerheads may suggest that thermosensitivity has a genetic basis, that certain genotypes confer differential fitness benefits to the sexes (such as maturation age, sexual size dimorphism, and offspring size) depending on hatching temperature, and or that sexes within a cohort develop differential allele frequencies as time progresses, among other possible explanations (Berry and Shine 1980; Schroeder et al. 2016). Our increased discovery of sex-specific genotypes using *Ch. mydas* as a reference for SNP calling over *Chr. picta* argues for the practical value of establishing genome assembly resources for target species of conservation priority over less informative de novo approaches to biodiversity studies.

Management strategies for sea turtle populations should maintain large population sizes by preserving natural habitats to facilitate outbreeding and prevent inbreeding depression (Frankham and Ralls 1998; Frankham 2005). Attention to genetic factors and continued development of molecular markers will focus conservation efforts on the most threatened populations. Our population genomic results can facilitate detection of adaptive genetic variation, where detection of islands of selection in noncoding regions may reveal regulatory networks for functional gene complexes of potential adaptive importance. The genome-wide landscape of millions of new polymorphic biomarkers invites downstream bioinformatics investigation within a comparative evolutionary framework. By integrating SNP population data with tissue- and condition-specific RNA-seq signatures of differential gene expression and by mapping functional pathways of candidate loci to reference genome assemblies of target species (Edwards et al. 2005; Shedlock et al. 2007; Janes et al. 2010), we anticipate that a more model-based and predictive versus reactive approach to investigating marine turtles and managing their wild populations can be realized in the 21st century.

## Supplementary Material

Supplementary data are available at *Genome Biology and Evolution* online.

## Supplementary Material

evz190_Supplementary_Data

## Notes

**Data deposition:** Original DNA sequence data for this project have been deposited at the NCBI Biosample website for DNA & RNA Resources under the SRA accession PRJNA559677 with sample-specific accession numbers SAMN12551088-SAMN12551132.
