# Supplementary material for: Sea Turtle Population Genomic Discovery: Global and Locus-Specific Signatures of Polymorphism, Selection, and Adaptive Potential
Source: Genome Biol Evol. 2019 Sep 4;11(10):2797–806. doi: 10.1093/gbe/evz190 (PMC6786478; doi:10.1093/gbe/evz190)
Supplement: evz190_Supplementary_Data [file evz190_supplementary_data.zip › SUPPLEMENTARY DATA FILES FOR FINAL PUBLICATION evz190/SuppFigures 1-3 Captions.pdf]

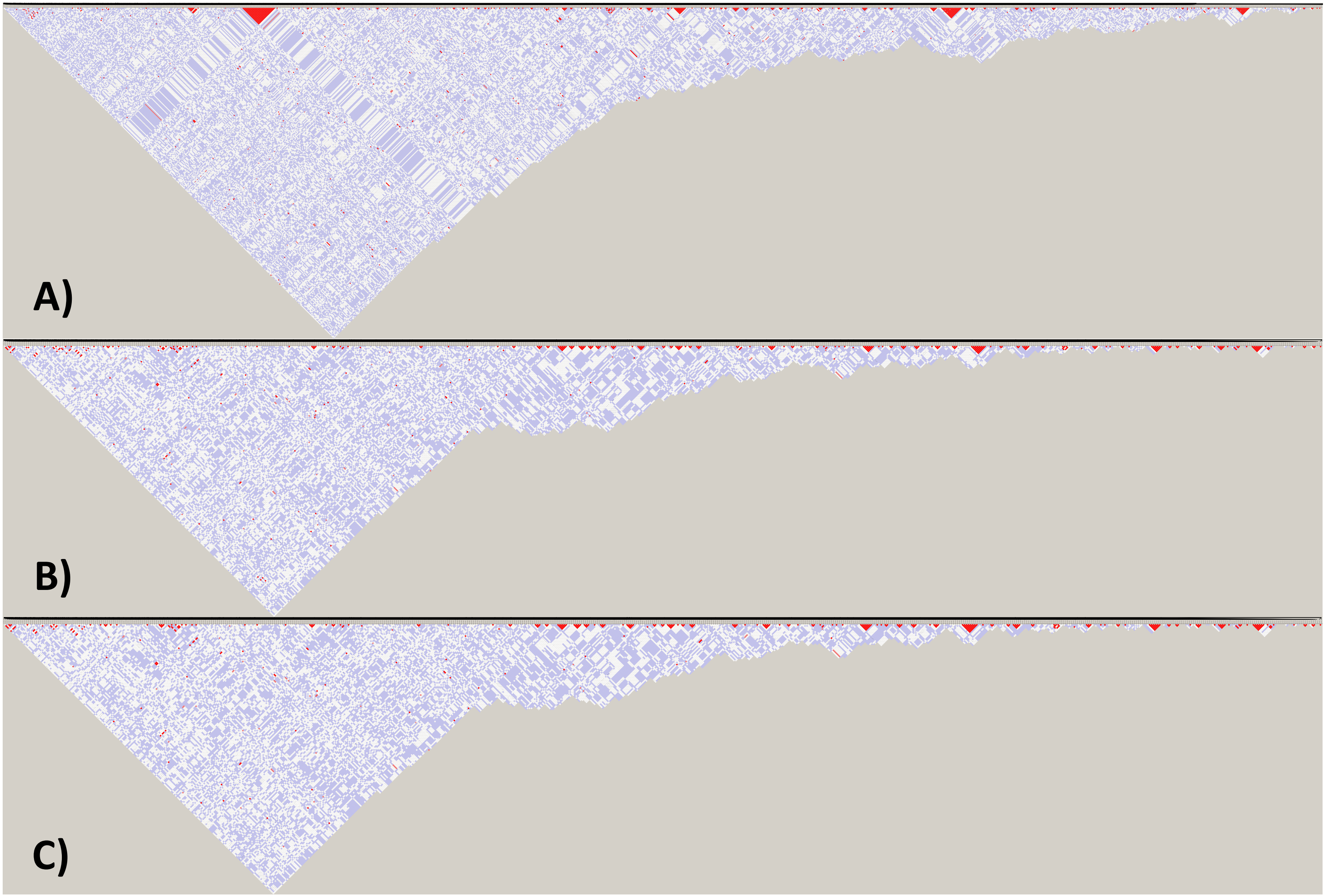

Supplementary Fig. 1 Linkage disequilibrium (LD) plots of loci produced via Haploview using *C. mydas* or *C. picta* as a reference during SNP calling and with consideration of Florida Bay (FB) or Atlantic (AC) loggerheads as a single group or two groups: **A)** *C.*

mydas, 1 group; **B)** *C. picta,* 1 group; **C)** *C. picta*, 2 groups. Color indicates D' and LOD values (white: D' < 1 and LOD < 2; blue: D' = 1 and LOD < 2; shades of pink/red: D' < 1 and LOD ≥ 2; bright red: D' = 1 and LOD ≥ 2). Numbers within squares represent D' * 100, and unnumbered squares have D' = 1.

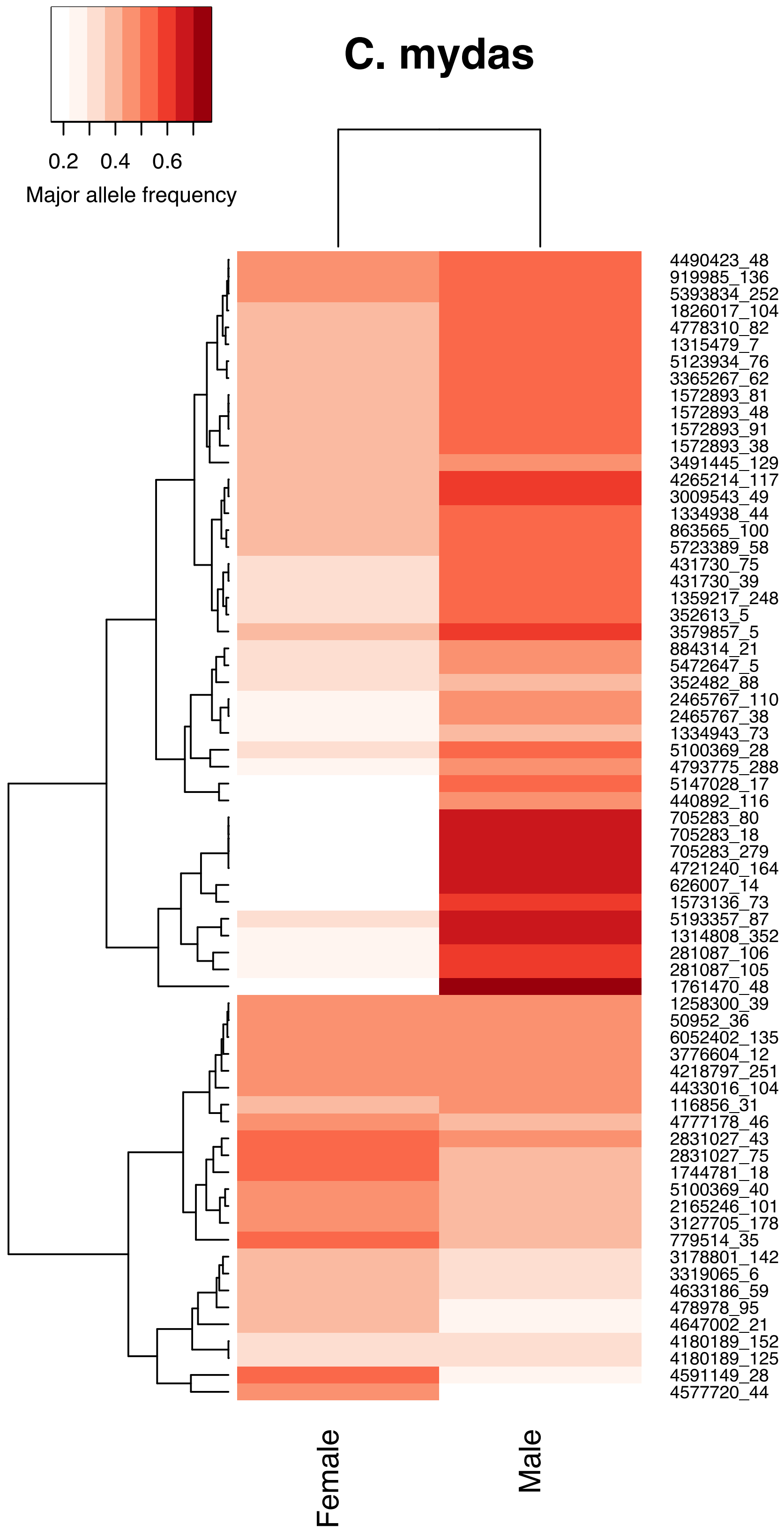
C. mydas
0.2
0.4
0.6
Major allele frequency
Female
Male
4490423_48
919985_136
5393834_252
1826017_104
4778310_82
1315479_7
5123934_76
3365267_62
1572893_81
1572893_48
1572893_91
1572893_38
3491445_129
4265214_117
3009543_49
1334938_44
863565_100
5723389_58
431730_75
431730_39
1359217_248
352613_5
3579857_5
884314_21
5472647_5
352482_88
2465767_110
2465767_38
1334943_73
5100369_28
4793775_288
5147028_17
440892_116
705283_80
705283_18
705283_279
4721240_164
626007_14
1573136_73
5193357_87
1314808_352
281087_106
281087_105
1761470_48
1258300_39
50952_36
6052402_135
3776604_12
4218797_251
4433016_104
116856_31
4777178_46
2831027_43
2831027_75
1744781_18
5100369_40
2165246_101
3127705_178
779514_35
3178801_142
3319065_6
4633186_59
478978_95
4647002_21
4180189_152
4180189_125
4591149_28
4577720_44

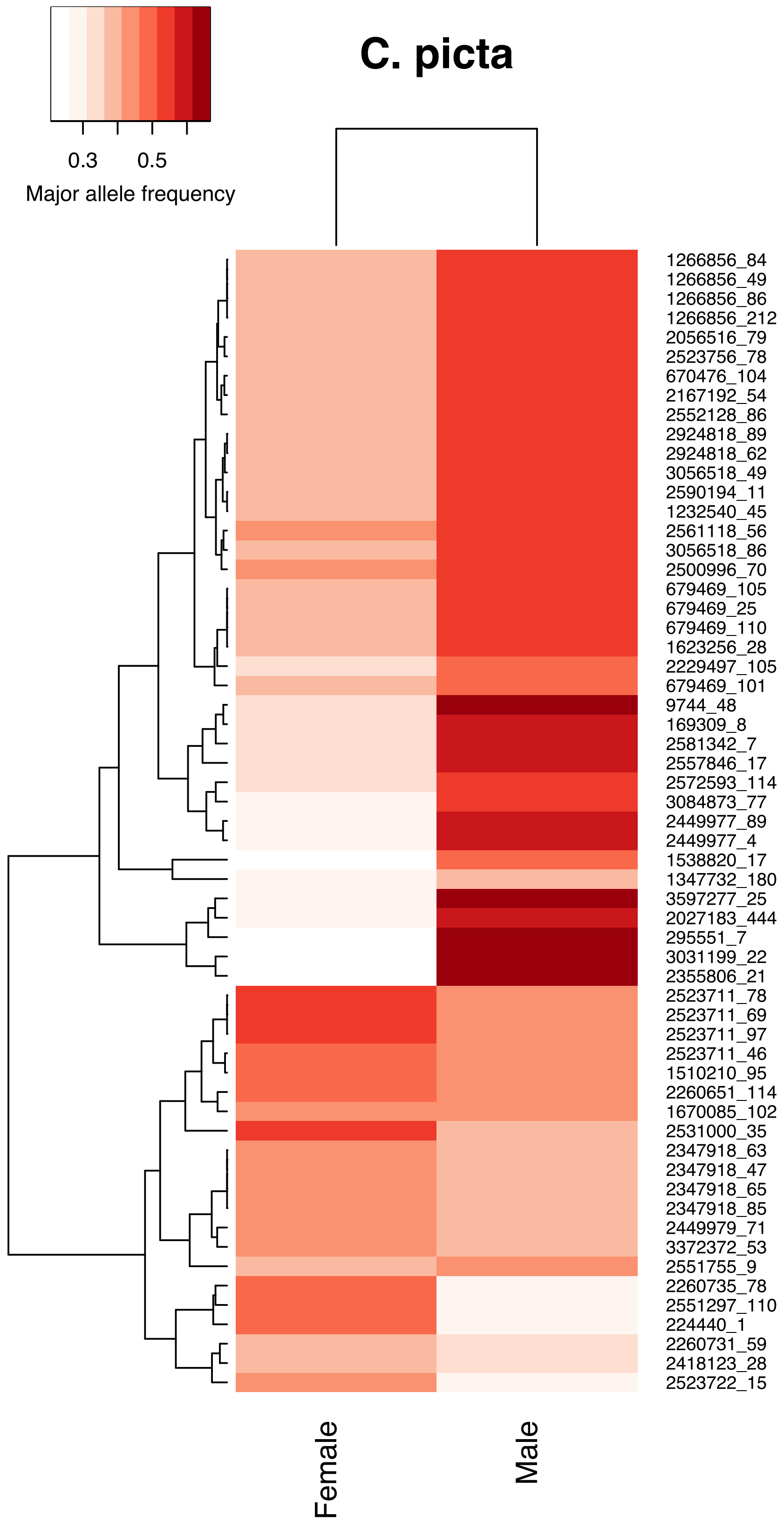
C. picta
0.3
0.5
Major allele frequency
1266856_84
1266856_49
1266856_86
1266856_212
2056516_79
2523756_78
670476_104
2167192_54
2552128_86
2924818_89
2924818_62
3056518_49
2590194_11
1232540_45
2561118_56
3056518_86
2500996_70
679469_105
679469_25
679469_110
1623256_28
2229497_105
679469_101
9744_48
169309_8
2581342_7
2557846_17
2572593_114
3084873_77
2449977_89
2449977_4
1538820_17
1347732_180
3597277_25
2027183_444
295551_7
3031199_22
2355806_21
2523711_78
2523711_69
2523711_97
2523711_46
1510210_95
2260651_114
1670085_102
2531000_35
2347918_63
2347918_47
2347918_65
2347918_85
2449979_71
3372372_53
2551755_9
2260735_78
2551297_110
224440_1
2260731_59
2418123_28
2523722_15
Female
Male

366

Supplementary Fig. 2 Heatmap of major allele frequencies of loci with sex-specific genotypes for male and female loggerheads, using *C. mydas* or *C. picta* as a reference during SNP discovery.

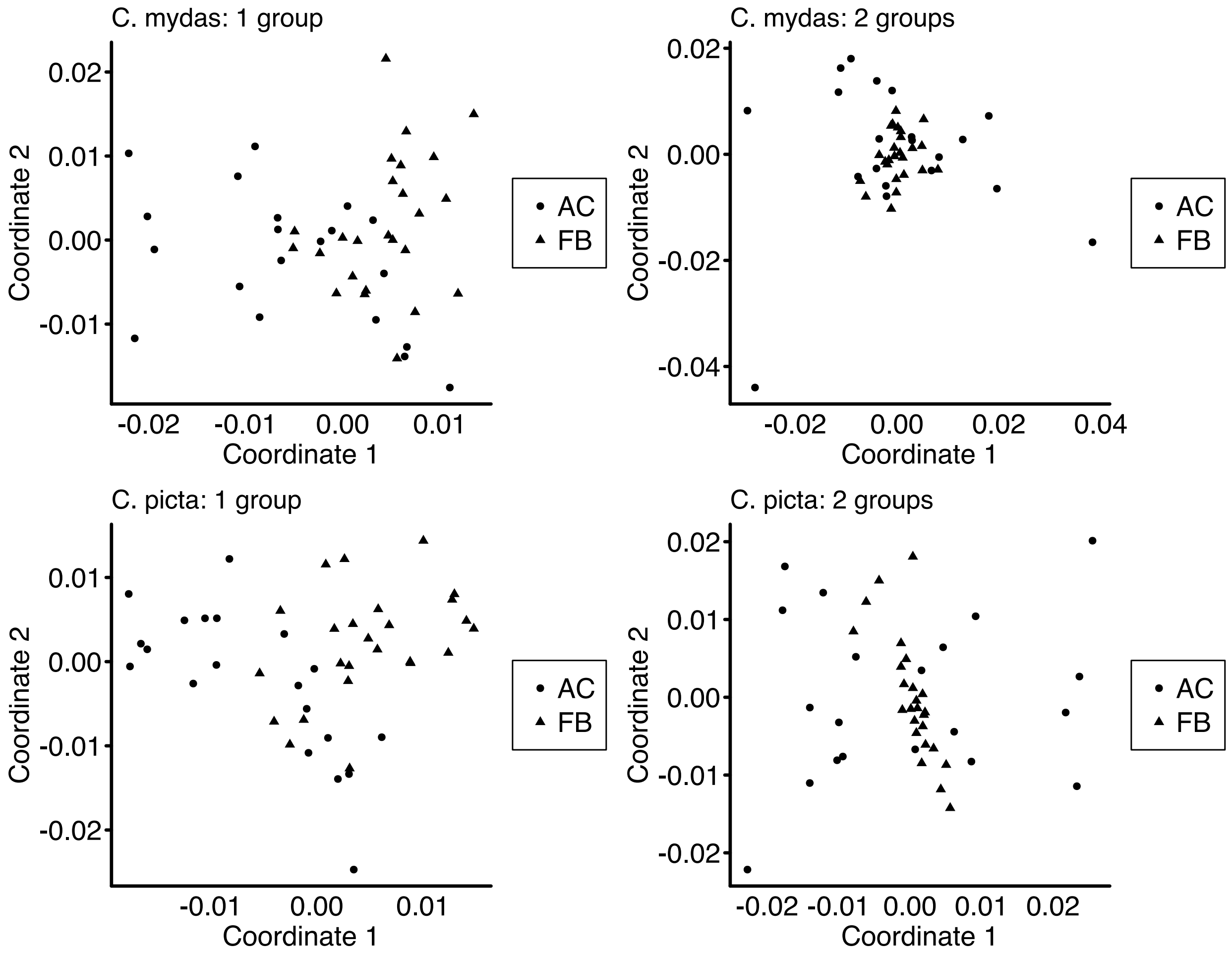

Supplementary Fig. 3 (MDS) plot. Estimated distribution of genetic distance among Florida Bay (FB) and Atlantic (AC) loggerhead sea turtles with consideration of AC and FB individuals as separate (2) or single (1) groups and using *C. mydas* or *C. picta* as a reference during SNP calling.
