# Supplementary figures and images for: Sea Turtle Population Genomic Discovery: Global and Locus-Specific Signatures of Polymorphism, Selection, and Adaptive Potential
Source: Genome Biol Evol. 2019 Sep 4;11(10):2797–806. doi: 10.1093/gbe/evz190 (PMC6786478; doi:10.1093/gbe/evz190)

# C. mydas

Major allele frequency

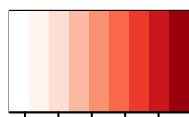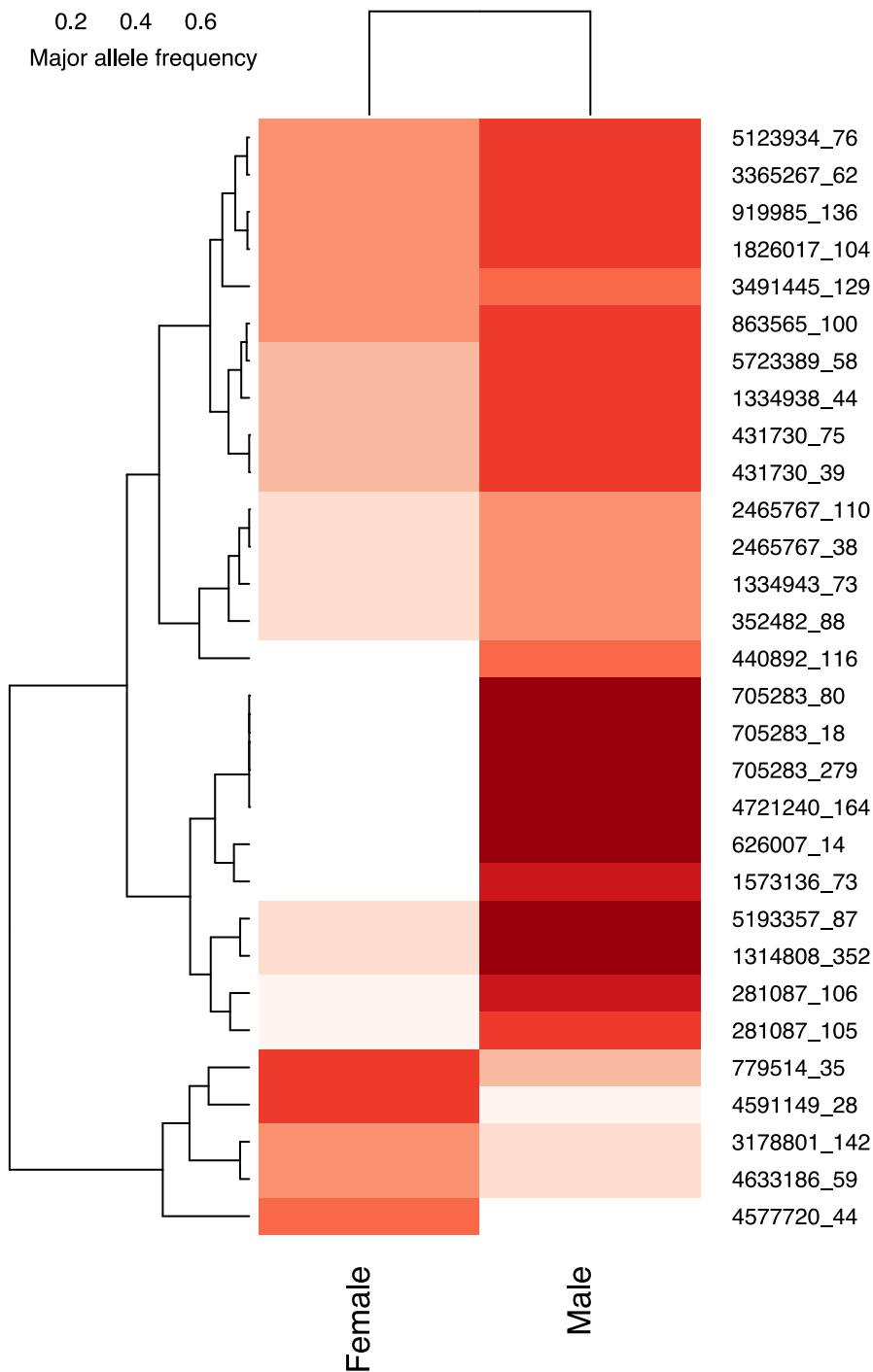

Supplement: evz190_Supplementary_Data [file evz190_supplementary_data.zip › SUPPLEMENTARY DATA FILES FOR FINAL PUBLICATION evz190/SuppFigure2_myd.pdf]

*C. mydas*: 1 group

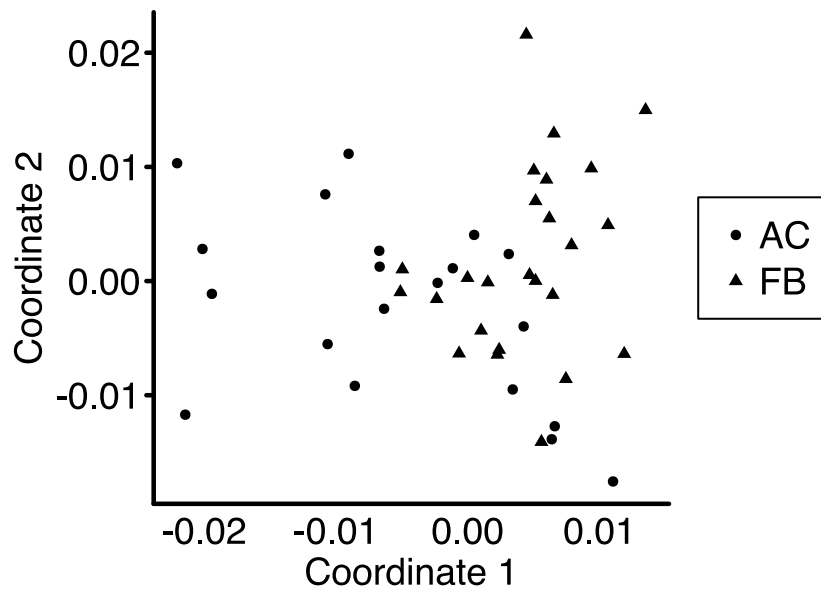

*C. mydas*: 2 groups

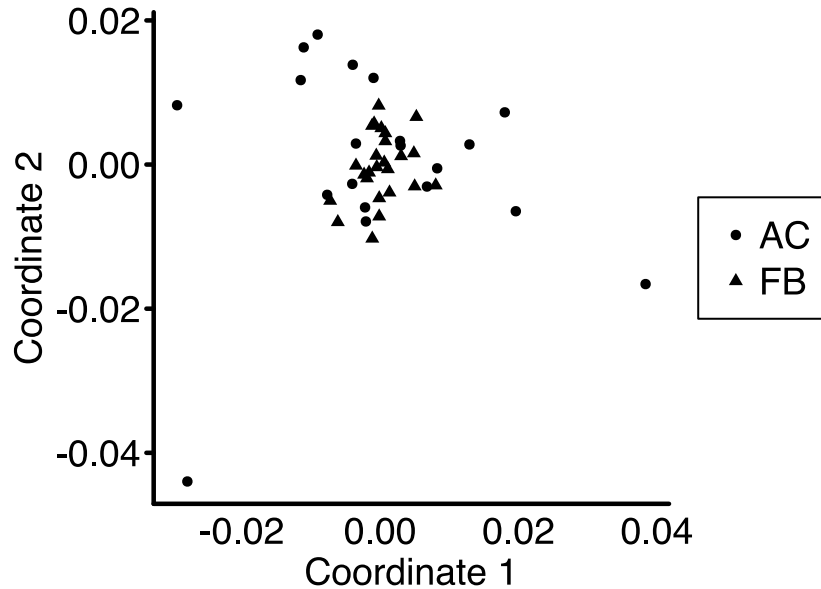

*C. picta*: 1 group

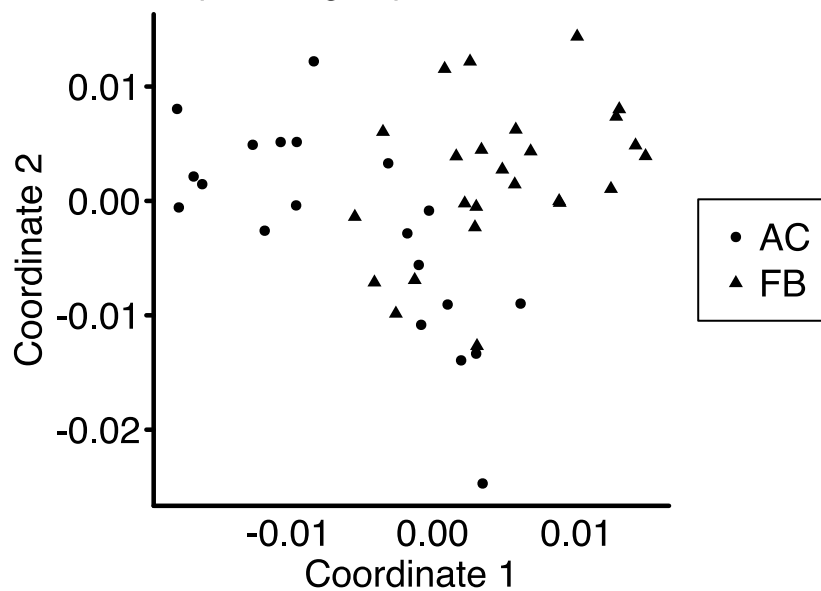

*C. picta*: 2 groups

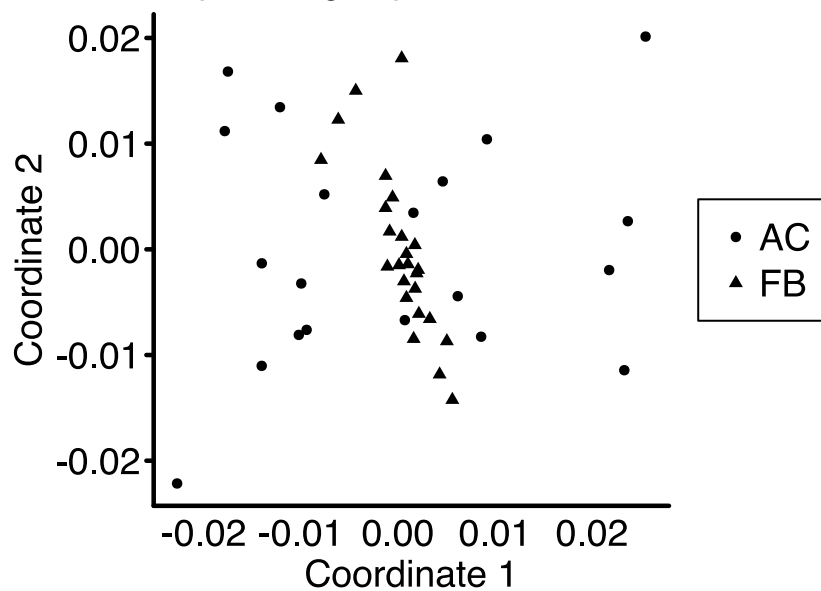

Supplement: evz190_Supplementary_Data [file evz190_supplementary_data.zip › SUPPLEMENTARY DATA FILES FOR FINAL PUBLICATION evz190/SuppFigure3.pdf]

# C. picta

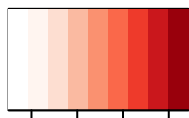

0.3 0.5

Major allele frequency

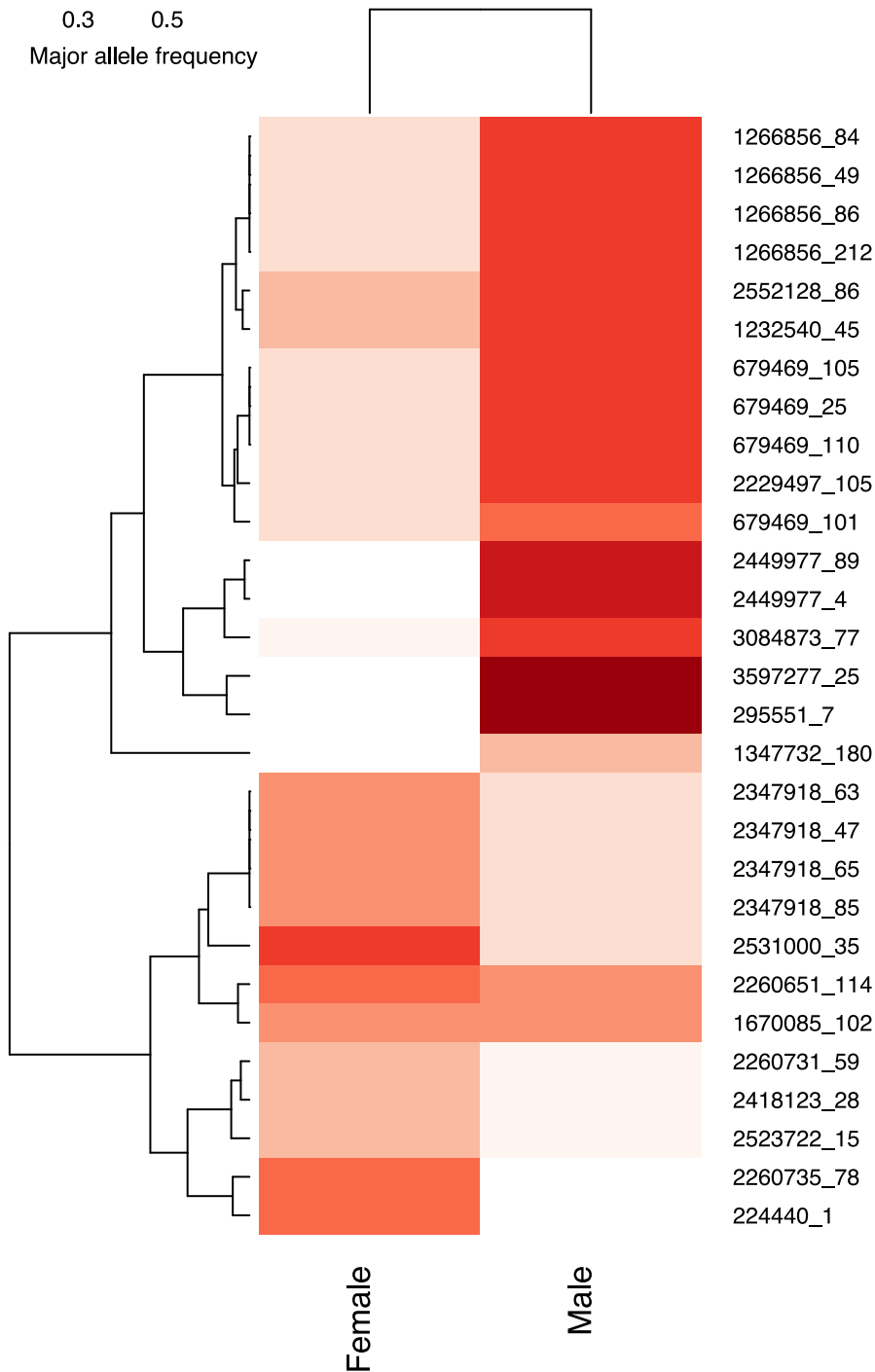

Supplement: evz190_Supplementary_Data [file evz190_supplementary_data.zip › SUPPLEMENTARY DATA FILES FOR FINAL PUBLICATION evz190/SuppFigure2_pic.pdf]
