# Supplementary material for: Sea Turtle Population Genomic Discovery: Global and Locus-Specific Signatures of Polymorphism, Selection, and Adaptive Potential
Source: Genome Biol Evol. 2019 Sep 4;11(10):2797–806. doi: 10.1093/gbe/evz190 (PMC6786478; doi:10.1093/gbe/evz190)

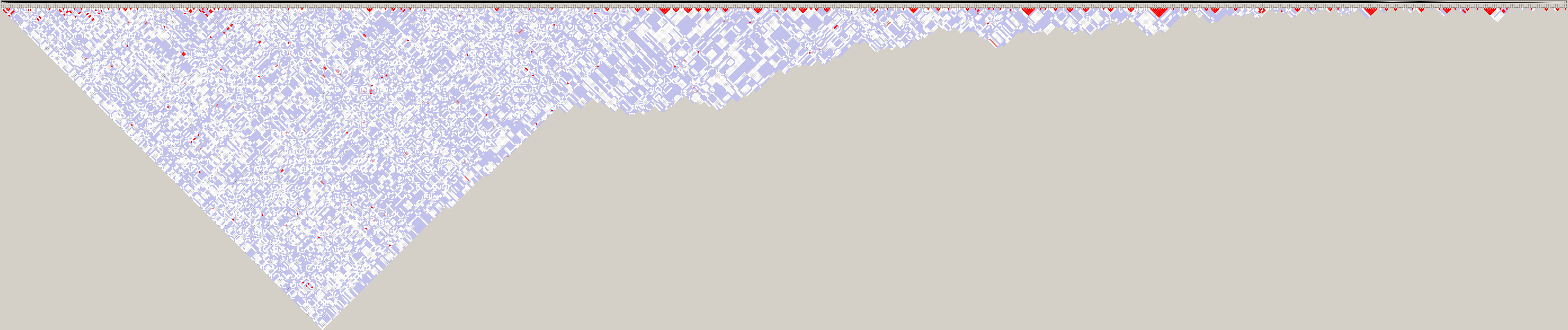

Supplement: evz190_Supplementary_Data [file SuppFigure1_pic1.pdf › SUPPLEMENTARY DATA FILES FOR FINAL PUBLICATION evz190/SuppFigure1_pic1.pdf]
